# The Impact of PEGylation on Cellular Uptake and In Vivo Biodistribution of Gold Nanoparticle MRI Contrast Agents

**DOI:** 10.3390/bioengineering9120766

**Published:** 2022-12-04

**Authors:** Nagwa El-Baz, Betty M. Nunn, Paula J. Bates, Martin G. O’Toole

**Affiliations:** 1 Department of Pharmacology and Toxicology, University of Louisville, Louisville, KY 40292, USA; 2 Department of Bioengineering, University of Louisville, Louisville, KY 40292, USA; 3 Department of Medicine, University of Louisville, Louisville, KY 40202, USA

**Keywords:** gold nanoparticle, AS1411, gadolinium chelate, polyethylene glycol, cancer-targeted, opsonization, protein corona, biodistribution

## Abstract

Gold nanoparticles (GNPs) have immense potential in biomedicine, but understanding their interactions with serum proteins is crucial as it could change their biological profile due to the formation of a protein corona, which could then affect their ultimate biodistribution in the body. Grafting GNPs with polyethylene glycol (PEG) is a widely used practice in research in order to decrease opsonization of the particles by serum proteins and to decrease particle uptake by the mononuclear phagocyte system. We investigated the impact of PEGylation on the formation of protein coronae and the subsequent uptake by macrophages and MDA-MB-231 cancer cells. Furthermore, we investigated the in vivo biodistribution in xenograft tumor-bearing mice using a library of 4 and 10 nm GNPs conjugated with a gadolinium chelate as MRI contrast agent, cancer-targeting aptamer AS1411 (or CRO control oligonucleotide), and with or without PEG molecules of different molecular weight (Mw: 1, 2, and 5 kDa). In vitro results showed that PEG failed to decrease the adsorption of proteins; moreover, the cellular uptake by macrophage cells was contingent on the different configurations of the aptamers and the length of the PEG chain. In vivo biodistribution studies showed that PEG increased the uptake by tumor cells for some GNPs, albeit it did not decrease the uptake of GNPs by macrophage-rich organs.

## 1. Introduction

In recent years, gold nanoparticles (GNPs) have grabbed the attention of many researchers in the biomedical field. Their unique physicochemical properties [1], ease of synthesis [2], and ability to be functionalized with different biological moieties make them promising candidates for many applications such as diagnostic agents [3,4,5,6,7], photothermal therapy [8,9,10], targeted drug delivery, and as drug carriers [11,12,13,14,15].

Once GNPs are administered into the bloodstream, they interact with abundant proteins in the blood circulation leading to the formation of protein coronae on their surfaces. Certain components in the protein corona, namely complement proteins and immunoglobulins (IgG), act as opsonin proteins. These in turn are recognized by complement and Fc receptors on the cell surface of immune cells [16,17] which then flag the particles for rapid clearance by the immune system thereby shortening their blood residence time and impacting their biological fate [17,18,19,20,21]. 

Different strategies have been developed to mitigate the process of opsonization on nanoparticles and attain longer blood circulation half-lives [22,23,24,25,26,27], including grafting GNPs with polyethylene glycol (PEGylation). The hydrophilic nature of PEG creates a hydrated sphere around the particles that sterically prevents the interaction of GNPs with each other, leading to increasing particle stability and preventing aggregation [16]. Moreover, PEGylation decreases the interaction of particles with opsonin proteins and decreases their susceptibility to be cleared by the immune system, providing longer blood circulating time for GNPs to be able to reach targeted tissues [28,29,30,31]. It has been reported that many factors can influence the relative opsonization and circulation time of PEGylated particles, such as PEG molecular weight [32,33], surface density [34], conformation of PEG [34], and the size and properties of the GNPs [35].

Confounding the issue, numerous studies have reported that PEGylation not only fails to decrease protein adsorption but that it in fact also increases protein binding [36,37]. Furthermore, some researchers have demonstrated that PEGylation shortens the circulating time upon administration of the second dose in what is known as the “accelerated blood clearance” (ABC) phenomenon due to the formation of anti-PEG IgM immunoglobulins—albeit the effect of ABC seems temporary and scant after a third dose of particles [38,39]. Moreover, some studies have shown that PEGylation has a negative effect on the uptake of the particles by tumor cells or other targeted cells due to its hydrophilic nature and nanoparticle shielding properties [33,36]. 

In a previous study, we reported the ability of a 4 nm cancer-targeted gold nanoparticle MRI contrast agent to selectively target cancer cells and provide high relaxivity enhancement upon MRI imaging [40]. The contrast agent consisted of GNPs conjugated with a gadolinium chelate and an AS1411 aptamer. AS1411 is a G-rich synthetic DNA oligonucleotide with a G quadruplex structure, which selectively targets cancer cells by binding to nucleolin protein that is expressed on the cell surface and in the cytoplasm of cancer cells [41,42,43]. 

Here in this study, we investigated the impact of PEGylation on cancer-targeted gold nanoparticle MRI contrast agents. GNPs of 4 nm and of 10 nm core size were conjugated with gadolinium chelate and AS1411 aptamer with and without PEG of different molecular weights (M_w_: 1, 2, and 5 kDa). We quantified the number of adsorbed proteins after incubation of the PEGylated and non-PEGylated particles in human serum and quantified the uptake of the different particles with and without protein corona by the human breast cancer cell line MDA-MB-231 and the murine monocyte/macrophage cell line RAW 264.7. In addition, we investigated the in vivo biodistribution of GNPs using the murine model of 4T1 mammary carcinoma in BALB/c female mice.

## 2. Materials and Methods

### 2.1. Materials

Gold (III) Chloride Trihydrate (HAuCL_4_·3H_2_O) was purchased from Alfa Aesar (Tewksbury, MA, USA). The 10 nm GNPs were purchased from Nanopartz (Salt Lake, UT, USA). Citric acid, trisodium salt (Na_3_C_6_H_5_O_7_), Sodium Borohydride (NaBH_4_), Gadolinium trichloride hexahydrate (GdCl_3_·6H_2_O), dithiothreitol (DTT), Sodium dodecyl sulfate (NaC_12_H_25_SO_4_), anhydrous sodium bicarbonate (NaHCO_3_), sodium phosphate monobasic dihydrate (NaH_2_PO_4_.2H_2_O), sodium phosphate dibasic (Na_2_HPO_4_), and human serum obtained from male AB plasma of multiple donors were all purchased from Sigma Aldrich (St. Louis, MO, USA). The 1,4,7,10-tetraazacyclododecane-1,4,7-tris(acetic acid)-10-(2-thioethyl) acetamide, C_18_H_33_N_5_O_7_S.2CF_3_CO_2_H (DO3ASH) was purchased from Macrocyclics, Inc. (Dallas, TX, USA). A 10.0X phosphate buffer saline (PBS, pH 7.4) and Pierce BCA protein assay kits were purchased from Thermo Fisher Scientific (Waltham, MA, USA). Methoxy polyethylene glycol thiol (mPEG-SH), MW 1, 2, and 5 kDa (PDI (polydispersity index) = 1.02–1.05 with very narrow MW distribution) were purchased from Creative PEGworks (Durham, NC, USA). Nanopure ultrapure water (Sartorius Arium, resistivity of 18.2 MΩ-cm) was used for preparing all aqueous solutions. Hydrochloric acid (HCl) and Nitric Acid (HNO_3_) were analytical grades and purchased from VWR (Radnor, PA, USA). Aqua regia solution (3 parts HCl and 1 part HNO_3_) was used to clean all glassware for GNP synthesis. Oligonucleotides (Oligo) having a regular DNA backbone (phosphodiester), a 5′-Thiol C6 S-S modification (Thio-MC6-D), a 5′-6T spacer (for AS1411 and CRO), and high-performance liquid chromatography (HPLC) purification were supplied by Integrated DNA Technologies (IDT) [Coralville, IA, USA]. The oligonucleotides sequences used were 5′-/5ThioMC6-D/TTT TTT GGT GGT GGT GGT TGT GGT GGT GGT GGT TT-3′ (AS1411), and 5′-/5ThioMC6-D/TTT TTT CCT CCT CCT CCT TCT CCT CCT CCT CCT TT-3 (CRO). A quantity of 0.5 Trypsin- EDTA (10X) was purchased from Thermo Fisher Scientific (Waltham, MA, USA). 

The MDA-MB-231 breast cancer cells, RAW 264.7 murine monocyte/macrophage cells and 4T1 (CRL-2539) cells were purchased from ATCC (Manassas, VA, USA). Dulbecco’s Modified Eagle Medium (DMEM) and RPMI media were purchased from Corning (Manassas, VA, USA). Fetal bovine serum (FBS) was purchased from Atlanta Biologicals (Flowery Branch, GA, USA). Penicillin–Streptomycin was purchased from Hyclone/Cytiva (Marlborough, MA, USA). 

UV absorption spectra were measured with the UV–Visible Spectrometer (Varian Cary 50 BIO UV, Agilent Technologies, Santa Clara, CA, USA). Dynamic Light Scattering measurements (DLS) and the Zeta potential measurements were acquired on latter samples using a NanoBrook Zeta PALS Zeta Potential Analyzer (Brookhaven Instruments, Holtsville, NY, USA). 

The concentrations of gold and gadolinium were analyzed by Agilent 7800 ICP-MS (Inductively Coupled Plasma Mass Spectrometry, Agilent Technologies Inc., Santa Clara, CA, USA). Before the sample assay, ICP-MS machine performance was checked by 1ppb tuning solution and the assay program was auto-tuned by 10 ppb tuning solution (Agilent Technologies, Cat# 5188-6564). Gd and Au standards were purchased from Agilent Technologies Inc. (Cat# ICP-064-25 and Cat# ICP-079) and diluted in serial concentration as 0, 1, 2, 5, 10, and 50 ng/mL by 2% nitric acid/DI water solution as standards. A 500 ppb internal standard solution (Agilent Technologies, Cat# 5188-6525) containing Bi, Ge, Ln, Li, Lu, Rh, Sc, and Tb was used to evaluate the recovery rate/machine operation condition. Samples were loaded by an Agilent SPS 4 Autosampler. 

### 2.2. GNP Synthesis

The 4 nm GNPs were synthesized according to the procedure described by Masitas and coworkers [44]. Briefly, a 2.5 mL of 0.01 M citric acid, trisodium salt was added to 95.0 mL of nanopure water under intense stirring. Then, 2.5 mL of 0.01 M HAuCl_4_ solution was added followed immediately by 3.0 mL of 0.1 M sodium borohydride at 4 °C. The solution was stirred for 2 h. GNP size was determined by UV–visible spectroscopy and dynamic light scattering [45]. The 10 nm GNPs were purchased as mentioned before.

### 2.3. Chemical Synthesis of Gadolinium (III) DO3A-SH (Gd)

A chelation process was performed between 1,4,7,10-tetraazacyclododecane-1,4,7-tris(acetic acid)-10-(2-thioethyl) acetamide, C_18_H_33_N_5_O_7_S.2CF_3_CO_2_H (DO3ASH) and GdCl_3_ by mixing an aqueous solution of DO3A-SH (17.1 mg in 200 uL nanopure water) with an aqueous solution of GdCl_3_ (22.23 mg in 200 uL nanopure water) at room temperature [46,47,48]. The reaction mixture pH was then adjusted to 6.0 by titrating 1.0 M sodium bicarbonate and the mixture was incubated overnight at 60 °C. During the reaction, the pH was monitored three times and bicarbonate solution was added as necessary to keep the pH in the range of 6–7. Afterward, the reaction mixture pH was adjusted to 9–10 using sodium bicarbonate and then centrifuged at 3000× *g* for 15 min. 

### 2.4. Annealing and Preparing of AS1411 and CRO for Conjugation to GNPs

Oligonucleotide solutions were prepared as previously reported [49]. Briefly, 500.0 µL of 500 µM oligonucleotide solution was prepared by suspending AS1411 and CRO in nanopure water. Before use, the disulfide protecting group on the oligonucleotide was cleaved with dithiothreitol (DTT). A 250.0 µL solution of 1M DTT was added to the oligonucleotide solution and heated to 90 °C for 1 h (0.1 M DTT, 0.18 M phosphate buffer (PB), pH 8.0). The cleaved oligonucleotides were purified using a NAP-25 column eluted with PB. Thereafter, the eluted solution of freshly cleaved oligonucleotides was added to gold nanoparticle dispersions.

### 2.5. Preparation of PEG Solution

Methoxy polyethylene glycol thiol (mPEG-SH), M_W_ 1, 2, and 5 kDa were suspended in nanopure water and filtered using a 0.2 um syringe filter before use.

### 2.6. Conjugation of Gd(III)-DO3A-SH (Gd), AS1411 and/or CRO, and mPEG to GNP

The 4 nm and 10 nm GNPs (~150 nM and ~10 nM for 4 and 10 nm, respectively) were functionalized with the MRI contrast agent Gd, either AS1411 or CRO, and with or without 1, 2, and 5 kDa mPEG chains [44]. All the functionalizing agents (Gd, AS1411, CRO, PEG (1, 2, and 5 kDa)) were bound directly to the surface of GNPs using a thiol–gold bond. The concentrations of the different agents were added × times the gold concentrations as shown in Table 1.

Sixteen different formulations of GNPs were synthesized and incubated at room temperature for 20 min and then sonicated for 10 min. The solutions were incubated overnight at 37 °C. Then, 10× PBS solution was added gradually over 5 days until the concentration of the GNP solutions reached 137 mM NaCl (during each salting time, the GNP solutions were sonicated for 10 min and followed by incubation at 37 °C). The excess oligonucleotides and Gd were removed via centrifugal filtration at 3000× *g* for 30 min. 

### 2.7. Quantification of AS1411 and CRO per Nanoparticle

A quantity of 20.0 μL of each GNP sample was added to 200 μL of 1.0 M DTT and 2.28 mL of PB (pH 8). The mixture was incubated for 24 h at 37 °C to cleave the gold oligonucleotide–thiol bond. The mixture was purified using a NAP-25 column to separate the oligos from DTT. Then, the released oligonucleotides were quantified using UV absorption spectra. 

### 2.8. Quantification of Gadolinium

A quantity of 20.0 μL of each GNPs sample was added to 800 μL of aqua regia and 600 μL of PBS solution and heated to 60 °C for 2 h. The sample was then diluted with nanopure water to 4% acid concentration and analyzed with ICP-MS.

### 2.9. Protein Quantification by Bicinchoninic Acid (BCA) Assay

A quantity of 1 mL of 4 nm GNPs or of 10 nm GNPs (100 μL and 20 μL, respectively) was mixed with 1 mL of 10% human serum (HS) and incubated for 24 h at 37 °C under continuous shaking. GNPs with a protein corona were then separated from the HS by centrifugation 2 times at 20,000× *g* for 1 h to remove the unbound proteins. Next, 5% of sodium dodecyl sulfate was added to the GNPs with a protein corona and the samples were heated for 5 min at 60 °C to isolate the tightly bound proteins from the GNPs. The samples were centrifuged and the supernatants were analyzed using the BCA protein assay kit (Pierce).

### 2.10. MDA-MB-231 and RAW 264.7 Cells Culture

MDA-MB-231 cells were cultured in DMEM media containing 10% FBS and 1% Penicillin–Streptomycin. RAW 264.7 cells were cultured in RPMI media containing 10% FBS and 1% Penicillin–Streptomycin. Both cell lines were subcultured using trypsin and incubated in a 5% CO_2_ incubator. 

### 2.11. Cellular Uptake

A 12-well plate was seeded with 100,000 MDA-MB-231 and RAW 264.6 cells per well and then cultured in complete formulated media (DMEM and RPMI, respectively) for 24 h. The media were removed and the cells were treated with 4 or 10 nm GNPs in media (without FBS) in the presence or absence of 10% HS for 90 min or 4 h. At the designated endpoints, the cells were washed 3 times using 1X PBS, collected, and then counted using a hemocytometer. The cells were digested with aqua regia at 60 °C for 2 h. Then, the samples were diluted with nanopure ultrapure water to reach 4% acid concentration and then analyzed by ICP-MS.

### 2.12. In Vivo Biodistribution Study

All animal experiments were performed under the approved protocol of The Institutional Animal Care and Use Committee (IACUC) of the University of Louisville, IACUC 22107. Female 4-week-old BALB/c mice were ordered from The Jackson Laboratory (Farmington, CT, USA). The mice were quarantined for one week before starting the experiments. The mice were injected by 4T1 cells (500,000 cell count in 100 μL of PBS with MgCl_2_, CaCl_2_, and glucose) via subcutaneous injection in the right flank. After 10 days, the tumors had grown to an average volume of ~300 mm^3^ and the mice were divided randomly into 8 groups (n = 6) and injected with 100 μL of either 4 nm GNP-Gd-AS1411, GNP-Gd-AS1411-PEG 1K, GNP-Gd-CRO, and GNP-Gd-CRO-PEG 1K or their 10 nm GNP analogs via intraperitoneal injection at a dose of 2 mg/ kg of oligonucleotides. The mice were sacrificed at 24 and 72 h post-injection. Following euthanasia, the tumors, livers, and spleens were collected. The organs were dried in the oven at 60 °C overnight (organs were weighed before and after drying). The dried organs were digested in aqua regia for 24 h and then the samples were centrifuged at 3000× *g* for 30 min and diluted with nanopure water to be prepared for ICP-MS analysis.

### 2.13. Statistical Analysis

An unpaired *t*-test, a one-way ANOVA, and a two-way ANOVA were performed for the obtained data using GraphPad Prism version 9.3.1 for Mac (Graphpad Software, San Diego, CA, USA, www.graphpad.com, accessed on 23 October 2022). Differences were considered statistically significant with *p* < 0.05.

## 3. Results

### 3.1. Synthesis and Characterization of GNPs

**Figure 1:**
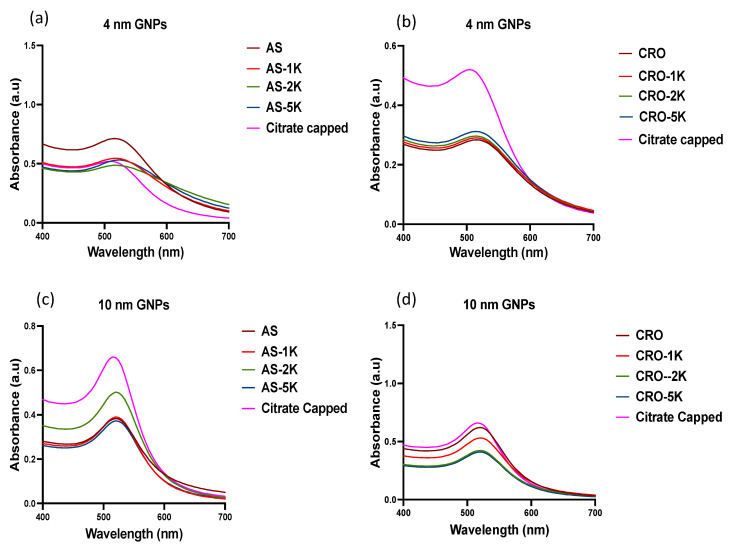
UV–vis spectra of GNPs; all particles conjugated with Gd. GNP formulations were denoted by the aptamer’s name CRO or AS (for AS1411) and PEG of different M_w_ (denoted as 1K, 2K, and 5K in the graph for 1, 2, and 5 kDa PEG, respectively). (**a**) Citrate-capped, non-PEGylated, and PEGylated 4 nm GNPs conjugated with AS1411. (**b**) Citrate-capped, non-PEGylated, and PEGylated 4 nm GNPs conjugated with CRO. (**c**) Citrate-capped, non-PEGylated, and PEGylated 10 nm GNPs conjugated with AS1411. (**d**) Citrate-capped, non-PEGylated, and PEGylated 10 nm GNPs conjugated with CRO.

Sixteen different formulations of MRI contrast agent GNPs candidates were synthesized with eight formulations for two GNP core sizes (4 and 10 nm). The GNPs were conjugated with Gadolinium (III) DO3A-SH (Gd), either AS1411 or CRO, and with or without PEG (M_W_: 1, 2, and 5 kDa). All the functionalizing agents (thiol-modified oligonucleotides, PEG-thiol, and DO3A-SH) were conjugated directly to the particle surface using thiol–gold bonding. The UV–vis absorption spectra showed surface plasmon resonance (SPR) peaks at 504 and 515 nm for citrate-capped 4 and 10 nm GNPs, respectively, while the conjugated 4 and 10 nm GNPs showed an average redshift in SPR to ~515 and ~520 nm, respectively (Figure 1). No significant broadening of UV–vis traces was noted, signifying successful conjugations with minimal aggregation of nanoparticles. In all figures in this manuscript, the GNP formulations were denoted by the aptamer’s name CRO or AS (for AS1411) and PEG of different M_w_ (denoted as 1K, 2K, and 5K for 1, 2, and 5 kDa PEG, respectively).

**Figure 2:**
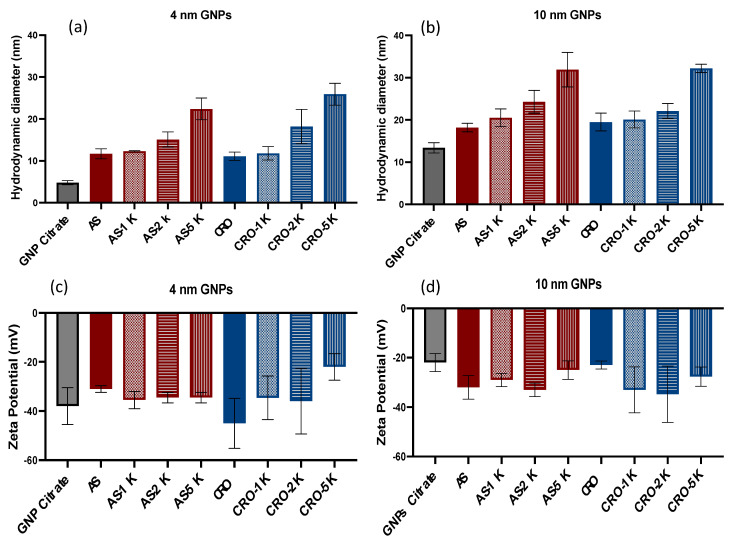
Characterization of GNPs. (**a**) Hydrodynamic diameters of 4 nm citrate-capped and conjugated GNPs and (**b**) the hydrodynamic diameters of 10 nm citrate-capped and conjugated GNPs. (**c**) ζ potential of 4 nm citrate-capped and conjugated GNPs. (**d**) ζ potential of 10 nm citrate-capped and conjugated GNPs. Each data point represents the mean ± standard deviation (SD) of triplicate measurments.

The average hydrodynamic diameters of 4 and 10 nm citrate-capped GNPs dispersed in water were 4.8 ± 0.5 and 13.4 ± 1.2 nm, respectively. The average hydrodynamic diameters of the conjugated GNPs were measured in 1× PBS and ranged from 11.1 to 25.9 nm and from 18.2 to 32.2 nm for 4 and 10 nm, respectively, Figure 2. These increases in size are consistent with previous research involving conjugation of AS1411 and CRO [14]. The particle sizes increased with increasing molecular weights of PEG. All GNPs were monodisperse with narrow size distribution and a PDI < 0.4. The citrate capping and other functionalizing agents on the GNPs conferred them negative zeta (ζ) potentials in PBS that ranged from −22 to −45 mV, implying that the applied coatings conferred stability in physiologically relevant levels of salt. The citrate capping and the negatively charged oligonucleotides conjugated to the GNPs conferred upon them negative zeta (ζ) potentials in PBS that ranged from −22 to −45 mV, implying that the applied coatings conferred stability in physiologically relevant levels of salt. In addition, we observed that the zeta potential of PEG 5 kDa GNPs are low in comparison to the other particles due to the large molecular weight of PEG, which covers the aptamers and masks their charges, Figure 2. 

The number of oligonucleotides (AS1411 and CRO) per GNP was quantified by UV–visible spectroscopy after cleaving the DNA from the particles using a DTT solution and column purification, Table 2. The number of Gd ions per nanoparticle was quantified by ICP-MS after nanoparticle digestion in Aqua Regia (Table 2). Both oligonucleotide and Gd ion numbers increase as the GNP core size increases from 4 to 10 nm and decrease as larger molecular weight PEG is added to the coating.

### 3.2. Number of Adsorbed Proteins on GNPs Surface

**Figure 3:**
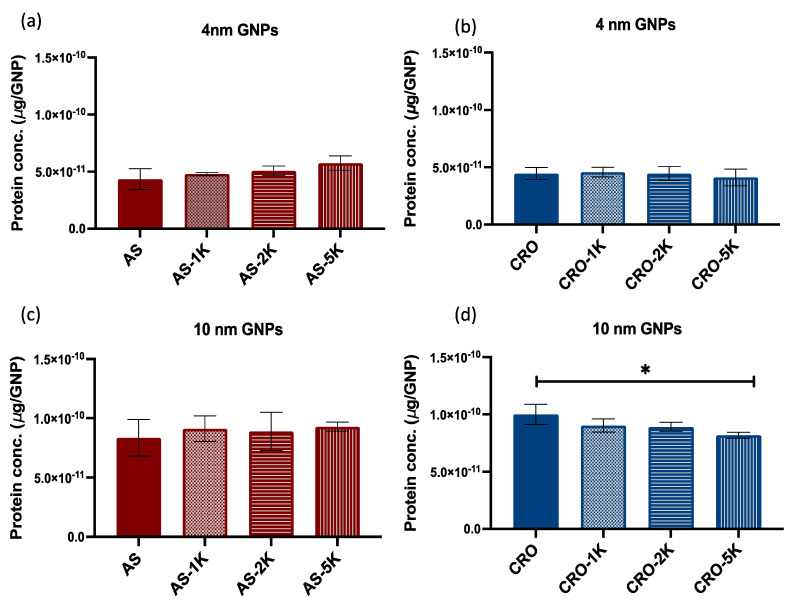
Quantification of the number of adsorbed proteins after incubation with 10% human serum. (**a**) 4 nm GNPs conjugated with AS1411. (**b**) 4 nm GNPs conjugated with CRO. (**c**) 10 nm GNPs conjugated with AS1411. (**d**) 10 nm GNPs conjugated with CRO. Each data point represents the mean ± standard deviation (SD) of triplicate experiments. * denotes *p* value < 0.05.

To quantify the number of adsorbed human serum proteins on PEGylated and non-PEGylated GNP surfaces, the GNPs were incubated in 10% human serum for 24 h at 37 °C. The GNPs were centrifuged 2 times to remove the unbound proteins and keep the GNPs with solid protein coronae only. Then, particles were treated with 5% SDS and heated to isolate the proteins followed by quantification of the proteins (μg of protein/GNP) with a BCA assay. The results suggested that 4 and 10 nm PEGylated GNP MRI contrast agents conjugated with AS1411 adsorbed greater numbers of serum proteins than did the corresponding non-PEGylated versions, but the differences between the protein numbers were not statistically significant. Moreover, we observed that the longer the chain of PEG the greater the number of proteins for the 4 nm GNPs, Figure 3a. The same pattern was not consistent with the 10 nm GNPs, but the GNPs with PEG M_W_ 5 kDa adsorbed the greatest number of proteins, Figure 3c.

**Figure 4:**
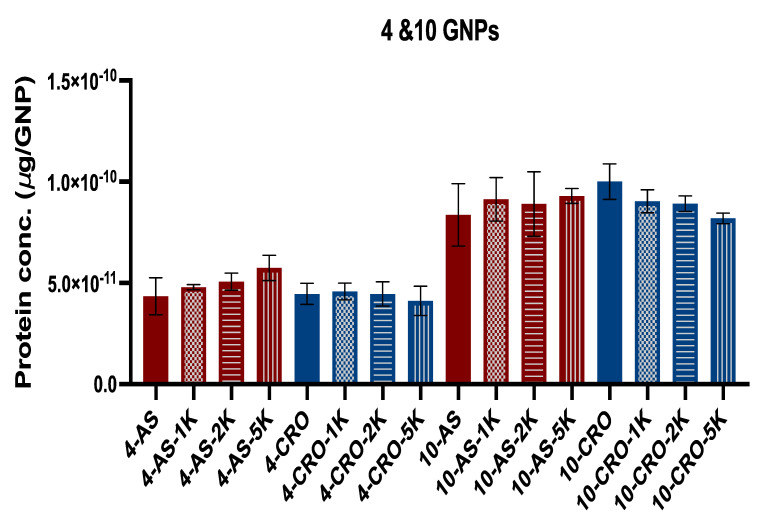
Comparison between 4 and 10 nm GNPs conjugated with AS1411/CRO indicates the numbers of adsorbed proteins on 10 nm GNPs are almost 2-fold the numbers found on 4 nm GNPs. Each data point represents the mean ± standard deviation (SD) of triplicate experiments.

In contrast to the above, PEGylation of the 4 and 10 nm MRI contrast GNPs conjugated with CRO mitigated the number of adsorbed proteins with the greatest reduction found with PEG M_W_ 5 kDa, Figure 3b,d (the reduction was significant with the 10 nm GNPs). We suggest that these dissimilarities between the adsorbed number of proteins on the PEGylated GNPs are attributed to the sequence and configuration of the aptamers. AS1411 is G-rich DNA, which forms a quadruplex 3D structure on the surface of GNPs, while CRO is C-rich linear DNA. The geometry of the DNA potentially affects the density and conformation of PEG chains on the GNP surface, thereby impacting the protein binding. Additionally, we noticed that the numbers of adsorbed proteins on the 10 nm GNPs are almost 2-fold the numbers found on the 4 nm GNPs, Figure 4. Considering that the 10 nm nanoparticle surface area is six times greater than that of the 4 nm particles, then smaller particles adsorbed a relatively greater number of proteins per unit surface area. Additionally, we compared the number of adsorbed proteins of all formulations of the 4 nm GNPs (conjugated with AS1411 or CRO) and found that the difference is not significant while the difference between all formulations of the 10 nm GNPs is significant (PEG M_W_ 5 kDa with CRO showed a significant reduction of the number of proteins). 

### 3.3. Cellular Uptake of GNPs

#### 3.3.1. Uptake by Macrophage Cells

**Figure 5:**
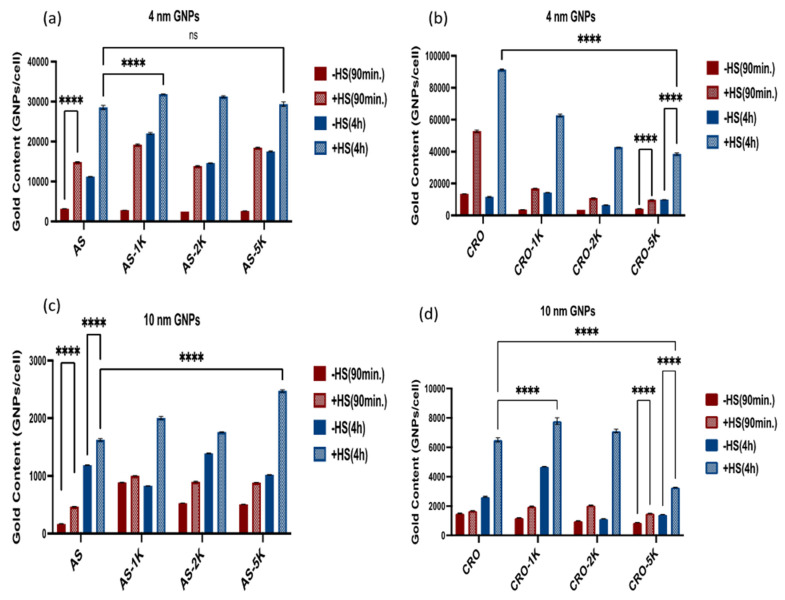
Cellular uptake of the GNP MRI contrast agents by RAW 264.7 cells at 90 min and 4 h, with 10% human serum-contained and human serum-free culture media. (**a**) The uptake of 4 nm GNP MRI contrast agents conjugated with AS1411. (**b**) The uptake of 4 nm GNP MRI contrast agents conjugated with CRO. (**c**) The uptake of 10 nm GNP MRI contrast agents conjugated with AS1411. (**d**) The uptake of 10 nm GNP MRI contrast agents conjugated with CRO. Each data point represents the mean ± standard deviation (SD) of triplicate experiments. **** denotes *p* < 0.0001 and ns denotes *p* non-significance.

Uptake studies were performed using RAW 264.7 macrophage cells since the complement receptors for the corresponding proteins in human serum are expressed on their surface. The cells were treated with the 16 different GNP MRI contrast agents in both 10% human serum-containing and human serum-free culture media for either 90 min or 4 h. We used a 30 nM concentration for 4 nm GNPs and normalized the concentrations of the 10 nm GNPs to have the same content of aptamer as the 4 nm GNPs. The cellular uptake of the particles was quantified by ICP-MS after cell digestion with aqua regia, Figure 5. 

In general, we found that the uptake of all GNPs in RAW 264.7 in the human serum-contained media was significantly higher compared with the uptake in human serum-free media, which indicates that the serum proteins impact and increase the uptake of particles by the macrophage cells. These results reinforce the notion that complement proteins in the protein corona are, in fact, recognized by macrophage complement receptors, thereby enhancing the uptake of particles by phagocytosis. Along with this, we observed that the uptake of GNPs is time-dependent, with higher amounts of gold being internalized at 4 h in comparison to 90 min. Additionally, all PEGylated 4 and 10 nm *GNPs conjugated with AS1411* have a significantly higher uptake than the non-PEGylated ones (except the 4 nm GNPs PEGylated with PEG 5kDa, which had a lower uptake than non-PEGylated ones—but the difference was not statistically significant), Figure 5a,c. Furthermore, the 4 nm GNPs showed a higher uptake than the 10 nm GNPs at the 4 h time point with serum-containing media. 

In contrast to the above, we observed a significant reduction in the uptake of the 4 nm PEGylated *GNPs conjugated with CRO* compared with the non-PEGylated CRO particles. In these experiments, the longer the chain of PEG, the lower the uptake by the cells. While the 10 nm PEGylated-1 kDa and 2 kDa GNPs had a higher uptake than non-PEGylated particles, the GNPs-CRO-PEG 5 kDa had the lowest uptake by macrophage cells, Figure 5b,d. We found that these results are correlated to the results of the BCA assay, as the PEGylated GNPs with AS1411 did not mitigate the number of adsorbed proteins while the PEGylated GNPs with CRO adsorbed fewer proteins than the non-PEGylated particles—although the difference is only significant with the 10 nm PEGylated-5kDa GNPs. 

Furthermore, we found that the uptake of the 4 and 10 nm *GNPs conjugated with CRO* is higher than the 4 and 10 nm GNPs conjugated with AS1411 at 4 h with human serum. This result could be related to the aptamer sequence and configuration as AS1411 forms a quadruplex structure while CRO is linear, which seemingly affects the cellular internalization.

#### 3.3.2. Uptake by Cancer Cells

**Figure 6:**
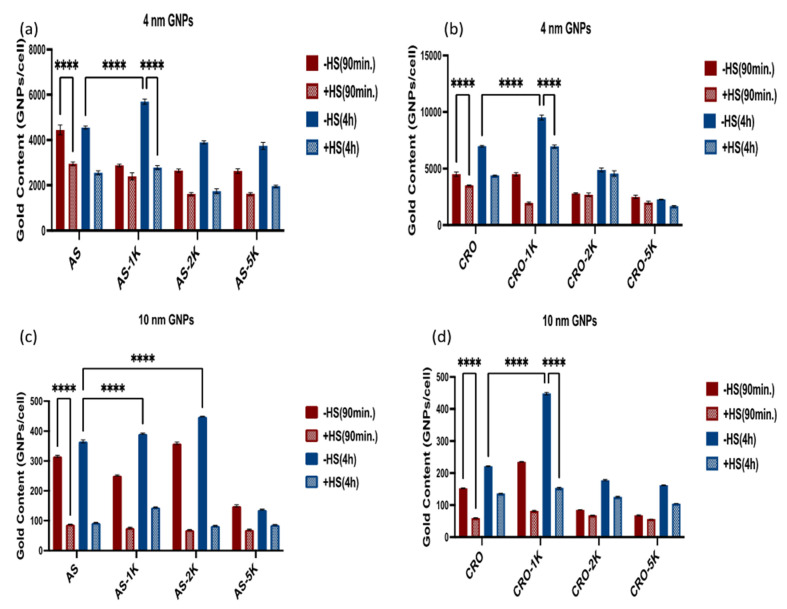
Cellular uptake of the GNP contrast agent by MDA-MB-231 cells with and without human serum after 90 min and 4 h. (**a**) The 4 nm group of GNPs conjugated with AS1411. (**b**) The 4 nm group of GNPs conjugated with CRO. (**c**) The 10 nm group of GNPs conjugated with AS1411. (**d**) The 10 nm group of GNPs conjugated with CRO. Each data point represents the mean ± standard deviation (SD) of triplicate experiments. **** denotes *p* < 0.0001.

MDA-MB-231 cells were subjected to the same nanoparticle treatments as RAW246.7, and the gold uptake amounts were quantified via ICP-MS. Contrary to RAW 264.7 cells, MDA-MB-231 cells that were treated with the GNP contrast agents in serum-containing media, showed significantly lower amounts of gold at the two time points compared with the samples in serum-free media, Figure 6, which suggests that serum proteins interfere with the nanoparticle uptake in cancer cells. Further, all 4 and 10 nm PEGylated 5 kDa GNPs exhibited the lowest uptake of gold regardless of the oligonucleotide, which differs from their uptake by macrophage cells. This reduction in uptake by PEG 5 kDa could be related to the high molecular weight, which covers the whole surface and covers the aptamers and increases the hydrophobicity of the particles. Additionally, shielding of the GNP zeta potentials by PEG-5 kDa could be another contributing factor. However, the uptake of 10 nm AS1411-GNPs with PEG-5 kDa was actually higher than the uptake for the lower MW PEG-coated GNPs. This suggests that several factors are contributing to the amount of cellular uptake by these nanoparticles. PEGylated 1 kDa nanoparticles (AS1411 and CRO) showed the highest uptake among all GNP contrast agents for the 4 and 10 nm GNPs conjugated with CRO and for the 4 nm GNPs conjugated with AS1411, Figure 6a,b,d. For the 10 nm GNPs conjugated with AS1411 (Figure 6c), the PEGylated 2 kDa uptake was significantly higher than the non-PEGylated and the PEGylated GNPs in the same group. Furthermore, we noticed that the uptake of all 4 nm GNPs is much higher than the uptake of all 10 nm GNPs implying that the uptake by cancer cells relied more on the gold core size than on the aptamer type or any other coating constituent.

### 3.4. In Vivo Biodistribution Study

**Figure 7:**
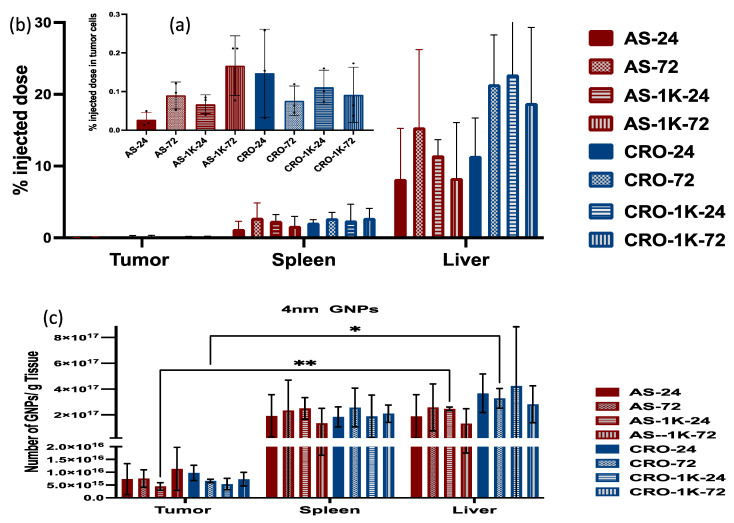
Quantification of 4 nm GNP accumulation in the tumor, liver, and spleen of mice that were injected with non-PEGylated and PEGylated (PEG Mw: 1 kDa) particles 24 and 72 h post-injection. (**a**) The percentage of the injected dose in tumor cells. (**b**) The percentage of the injected dose in the liver and spleen. (**c**) The number of GNPs/g tissue. Each data point represents the mean ± standard deviation (SD) of triplicate experiments. * denotes *p* < 0.05 and ** denotes *p* < 0.01.

**Figure 8:**
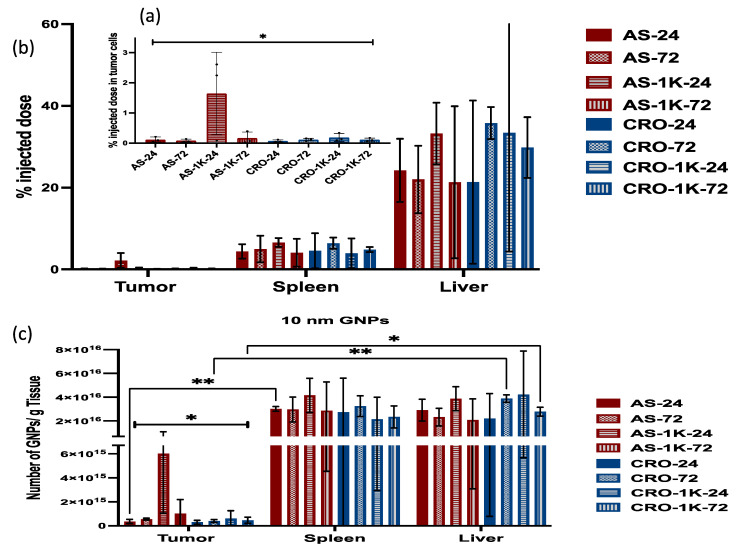
Quantification of 10 nm GNP accumulation in the tumor, liver, and spleen of mice that were injected with non-PEGylated and PEGylated (PEG Mw: 1 kDa) particles 24 and 72 h post-injection. (**a**) The percentage of the injected dose in tumor cells. (**b**) The percentage of the injected dose in the liver and spleen. (**c**) The number of GNPs/g tissue. Each data point represents the mean ± standard deviation (SD) of triplicate experiments. * denotes *p* < 0.05 and ** denotes *p* < 0.01.

An in vivo biodistribution study was conducted to better assess the differential uptake of the various GNP formulations by in situ tumor cells and macrophages functioning within an active immune system. A murine model of 4T1 mammary carcinoma in BALB/c female mice was used. When the tumors reached ~300 mm^3^, the mice were randomly divided into eight groups and injected via intraperitoneal injection with the GNPs. We excluded the PEGylated particles with PEG 2 and 5 kDa since they showed the lowest uptake by cancer cells in in vitro studies. The mice were euthanized after 24 and 72 h post-injection, and the tumors, livers, and spleens were collected for ICP-MS analysis. Results showed that the uptake of most of the PEGylated and non-PEGylated GNPs by the liver and spleen is significantly higher than their uptake by tumor cells, Figure 7 and Figure 8. The 4 nm PEGylated GNPs/AS1411 exhibited the highest uptake by tumor cells at 72 h, but the difference is non-significant among the means. The 4 nm non-PEGylated GNPs/CRO showed the highest uptake by tumor cells at 24 h and the difference is non-significant among the means as well. The uptake of all types of the 4 nm GNPs by the liver and spleen did not show any significance among their means. 

The uptake of the 10 nm PEGylated GNPs/AS1411 by tumor cells was significantly higher than the other particles at 24 h (*p =* 0.0162). 10 nm PEGylated GNPs/CRO displayed a slight increase compared with the non-PEGylated ones although the difference is non-significant. The results of the in vivo study revealed that PEGylation increases the uptake by tumor cells (especially 10 nm GNPs) for particles with AS1411, and in general, we observed that the uptake of large particles (10 nm) by the liver and the spleen is higher than the uptake of small ones (4 nm).

## 4. Discussion

In this study, we have demonstrated the synthesis and the characterization of a series of GNP-based MRI contrast agents using two sizes of GNPs (4 and 10 nm) fabricated with a gadolinium chelate, either AS1411 or CRO oligonucleotides, and with or without PEG 1, 2, and 5 kDa. UV–vis absorption spectra, DLS, and zeta potential were acquired and reported.

The protein corona that formed around the nanoparticles was purified from the GNPs and quantified by a BCA assay after incubation of the particles in 10% human serum for 24 h. PEGylation had an increased amount of protein binding with all the GNPs containing AS1411, while it decreased protein binding to the GNPs containing the CRO oligonucleotide. The role of PEG in protein adsorption is controversial in the literature, where many studies have shown that PEG provides a steric barrier that mitigates protein binding to nanoparticles [32,35] while others have reported that some proteins bind to PEGylated particles that do not adsorb to non-PEGylated particles [50,51]. Here, we suggest that the surface chemistry of GNPs plays a crucial role in the ability of PEG to impact protein corona formation, as the difference in the DNA sequence and the length of PEG together dictate the number of adsorbed proteins [52,53]. In addition, the density/conformation of PEG has been reported as an important factor, along with the gold core size, for determining the number of proteins bound to the nanoparticle [35,36,54,55]. Additionally, we have shown that the size of the particles affects the number of bound proteins, as smaller GNPs adsorbed relatively more proteins/surface area in comparison with the larger ones. This finding is supported by other reported studies [56].

The cellular uptake studies probed the impact of protein corona formation in the interaction of GNPs with macrophages and cancer cells. Serum proteins, including complement proteins, enhanced the uptake by macrophage cells due to recognition by complement receptors on the surface of macrophage cells. We demonstrated that the PEGylated GNPs/CRO reduced the uptake by macrophage cells with the highest reduction seen with PEG 5kDa, and that the internalization of smaller-sized particles was slightly higher than that of larger-sized ones [29,57]. While we noted that PEG did not mitigate the uptake of GNPs/AS1411, and indeed the highest uptake was with human serum-containing media, such behavior is also seen in the other studies, where it is reported that PEG activates the whole complement system, leading to the formation of SC5b-9 complex (the endpoint of complement activation) [58,59]. In contrast, serum proteins reduced the uptake of GNPs by cancer cells compared with the uptake of GNPs in serum-free media. More interestingly, there was a reduction in the uptake of larger particles in comparison with the uptake of smaller ones by at least 15, and 20-fold for GNPs/AS1411 and GNPs/CRO, respectively. These results are bolstered by many researchers [60,61,62,63,64,65], as it has been reported that the protein corona strongly reduces the adhesion of GNPs to the cell surface and hence decreases GNP-uptake efficiency. Others also documented that the adsorbed proteins compete with the GNPs and prevent their interaction with cell surface.

The in vivo study investigated the uptake of our candidate contrast agents in a murine xenograft tumor model. PEG significantly increased the uptake of 10 nm GNPs/AS1411 by tumor cells while the increase in uptake was non-significant for 4 nm. The uptake by macrophage-rich organs (liver and spleen) was significantly higher than the uptake by tumor cells, which seems to be a fundamental challenge for using GNPs in biomedicine. The physicochemical properties of GNPs (e.g., shape, diameter, surface charge, surface loading, and hydrophobicity) dictate their interaction with the immune system [66,67,68]. Furthermore, the different routes of GNP administration (e.g., intravenous, intradermal, nasal, subcutaneous, intraperitoneal, and inhalation) can evoke different immune responses [69,70,71]. In future studies, we will use GNPs with different densities of PEG and AS1411 to investigate which formula attains the highest uptake by tumor cells, and scan the live animals by MRI after injection of the GNPs at different time points.

## Figures and Tables

**Table 1:** Demonstration of the concentration amounts of functionalizing agents.

GNP	Gd	AS1411/CRO	PEG
4 nm non-PEGylated	10×	15×	-
4 nm PEGylated	6×	10×	6×
10 nm non-PEGylated	120×	90×	-
10 nm PEGylated	72×	60×	36×

**Table 2:** Quantification of oligonucleotides (AS1411/ CRO) and Gd per GNP.

Sample	Oligonucleotide/ 4 nm GNPs	Oligonucleotide/ 10 nm GNPs	Gd/ 4 nm GNPs	Gd/ 10 nm GNPs
GNP-Gd-AS1411	10	43.2	6.5	77.2
GNP-Gd-AS1411-PEG 1K	7.4	43.7	5.7	64.7
GNP-Gd-AS1411-PEG 2K	7.6	46.8	5.9	55.1
GNP-Gd-AS1411-PEG 5K	7	31.5	6.5	72.6
GNP-Gd-CRO	12.3	44.9	9.0	150.0
GNP-Gd-CRO-PEG 1K	6.3	42.9	7.9	81.8
GNP-Gd-CRO-PEG 2K	8.3	38.9	6.4	115.2
GNP-Gd-CRO-PEG 5K	8.7	40.7	7.9	113.3

## Data Availability

Not Applicable.
